# Coix Seed Oil Alleviates Hyperuricemia in Mice by Ameliorating Oxidative Stress and Intestinal Microbial Composition

**DOI:** 10.3390/nu17101679

**Published:** 2025-05-15

**Authors:** Guozhen Wu, Xinming Wang, Hongjing Dong, Jinqian Yu, Tao Li, Xiao Wang

**Affiliations:** 1 Shandong Engineering Research Center for Innovation and Application of General Technology for Separation of Natural Products, Shandong Analysis and Test Center, Qilu University of Technology (Shandong Academy of Sciences), Jinan 250014, China; 2 School of Pharmaceutical Sciences, Shandong University of Traditional Chinese Medicine, Jinan 250355, China; 3 Institute of Chinese Materia Medica Chemistry, Shandong Academy of Chinese Medicine, Jinan 250014, China; 4 Key Laboratory for Natural Active Pharmaceutical Constituents Research in Universities of Shandong Province, School of Pharmaceutical Sciences, Qilu University of Technology (Shandong Academy of Sciences), Jinan 250014, China

**Keywords:** coix seed oil, hyperuricemia, oxidative stress, gut microbiota, lipid metabolism

## Abstract

**Background**: Coix seed oil (YRO), rich in unsaturated fatty acids, has emerged as a promising intervention for hyperuricemia (HUA) due to its potential to alleviate oxidative damage and support organ health. **Methods**: The fatty acid composition of YRO was determined by gas chromatography–mass spectrometry (GC-MS). A HUA mouse model was established, and serum markers and hepatic enzymes were evaluated. Renal mitochondrial function was assessed using immunohistochemistry and immunofluorescence, and urate transporter expression, along with key signaling proteins, was quantified by Western blot analysis. Additionally, gut microbiota composition was analyzed, and non-targeted metabolomics was performed to observe alterations in serum lipid metabolites. **Results**: YRO significantly reduced serum uric acid (UA) levels and normalized hepatic enzyme activities. Histological evaluation revealed less tissue damage in both the kidney and the intestine. In the kidney, YRO improved mitochondrial function and supported antioxidant defenses via regulation of Keap1/Nrf2 signaling. In the intestine, YRO enhanced barrier integrity by increasing ZO-1, Occludin, and Claudin-1 expression. Moreover, YRO modulated gut microbiota by increasing beneficial bacteria (*Muribaculaceae*, *Prevotellaceae UCG-001*, *Lachnospiraceae_ NK4A136_group*, *Akkermansia*) while suppressing harmful species (*Bacteroides*, *Dubosiella*). Lipid metabolomics indicated a restoration of phospholipid balance through modulation of the PI3K/AKT/mTOR pathway. **Conclusions**: YRO supported metabolic health by promoting UA homeostasis, enhancing mitochondrial function, reinforcing antioxidant capacity, and maintaining gut integrity. These findings suggest that coix seed oil could serve as a nutritional supplement in managing HUA and related metabolic disturbances.

## 1. Introduction

Hyperuricemia (HUA) is a common metabolic disorder marked by elevated serum uric acid (UA) levels [1]. It results from excessive UA production or impaired renal and intestinal excretion [2]. Recent studies indicate that HUA is the second most prevalent metabolic disorder after diabetes, raising significant public health concerns [3]. Persistent UA accumulation leads to oxidative stress, inflammation, and organ dysfunction, contributing to gout, renal dysfunction, and metabolic syndrome [4,5]. As diet plays a crucial role in UA metabolism, exploring food-based approaches for HUA management has gained increasing attention.

Coix seed (*Coix lacryma-jobi* L.), known as Yi Yiren in Chinese, has long been incorporated into daily diets in East Asia for its nutritional and functional properties [6]. It is rich in bioactive compounds and has been consumed for centuries as a traditional dietary supplement with potential health benefits. Emerging evidence indicated that coix seed might contribute to UA regulation, yet its key bioactive components and precise mechanisms remain unclear [7]. Coix seed oil, a major component of coix seed, is particularly rich in unsaturated fatty acids (UFAs), which are known to support oxidative balance and organ protection [8,9]. Furthermore, recent studies demonstrated that functional oils could significantly modulate gut microbiota composition and metabolic pathways, influencing immune responses and physiological homeostasis [10,11]. Given these properties, coix seed oil holds promise for the development of functional food products targeting HUA management.

The kidney is the primary organ responsible for UA excretion and exhibits high metabolic activity with a dense mitochondrial network [12]. Excessive UA accumulation disrupts mitochondrial homeostasis, leading to elevated reactive oxygen species production, which further exacerbates cellular damage [13]. The Keap1/Nrf2 pathway serves as a defense system by activating antioxidant enzymes such as HO-1 and NQO-1, protecting cells from UA-induced oxidative injury [14,15]. When renal UA excretion is impaired, compensatory mechanisms enhance the elimination of intestinal UA [16]. However, this process often disrupts gut microbiota composition and reduces short-chain fatty acids (SCFAs) production, further affecting UA metabolism [17,18]. Importantly, UA homeostasis is intricately intertwined with broader metabolic networks. Recent studies highlighted its close linkage to lipid metabolism [19]. For instance, abnormal glycerophospholipid levels not only impair renal tubular function but also interfere with UA transport mechanisms, potentially exacerbating systemic metabolic dysregulation [20].

This study investigates the mechanisms of YRO in HUA management by analyzing renal and intestinal tissues, gut microbiota, and lipid metabolites. It aims to establish a foundation for future research on YRO as a functional food ingredient with potential applications in metabolic health.

## 2. Materials and Methods

### 2.1. Materials and Reagents

Coix seeds were purchased from BWT Chinese Herbal Medicine Drinks Slice Co., Ltd. (Jinan, China). Potassium oxonate (PO) was obtained from Shanghai Macklin Biochemical Technology Co., Ltd. (Shanghai, China). Hypoxanthine (HX) and sodium carboxymethyl cellulose (CMC) were supplied by Shanghai Yuanye Bio-Technology Co., Ltd. (Shanghai, China). Detailed information on antibodies used in this study was provided in Table S1.

### 2.2. Extraction, Esterification, and GC-MS Analysis of Fatty Acids from YRO

YRO was extracted and esterified according to previously established methods with modifications [21,22]. Briefly, dried coix seeds were ground into powders and subjected to ultrasonic-assisted extraction using petroleum ether as the solvent. The extraction was performed twice at room temperature, with each cycle lasting 2 h. The combined extracts were filtered and concentrated under reduced pressure to obtain YRO. For methyl esterification, the YRO was treated with a sulfuric acid-methanol solution (2:98, *v*/*v*) and heated in a 70 °C water bath for 60 min. After cooling, the reaction mixture was extracted with n-hexane to isolate the esterified compounds. The n-hexane layer was dried over anhydrous sodium sulfate, followed by filtration through a 0.45 μm membrane to yield the methyl-esterified YRO sample.

The composition of the fatty acid methyl esters was analyzed by gas chromatography-mass spectrometry (GC-MS) on a Shimadzu QP2020NX system (Kyoto, Japan) equipped with a quadrupole mass spectrometer and a DB5 column (30 m × 0.25 mm, 0.25 μm). The initial column temperature was set at 50 °C for 2 min, then increased at a rate of 6 °C/min to 250 °C, where it was held for 15 min. Helium was used as the carrier gas, with the injector temperature maintained at 250 °C and a split ratio of 1:30.

### 2.3. Animal Model and Experimental Design

**Figure 1:**
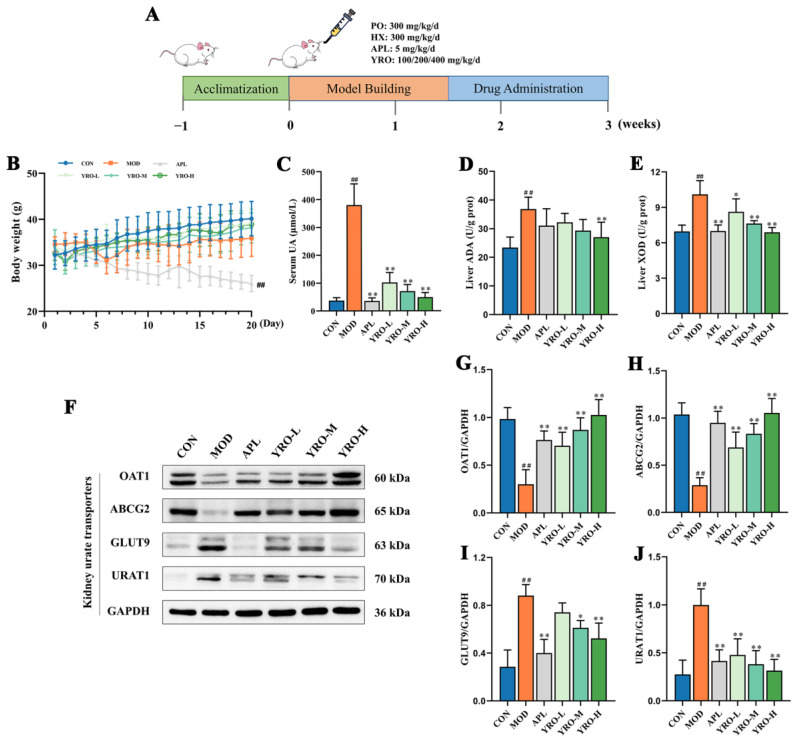
YRO alleviated HUA by improving UA metabolism. (**A**) Experimental scheme of animal procedures; (**B**) Changes in body weight during the experimental period; (**C**) Serum UA levels; (**D**) Hepatic ADA and (**E**) XOD activities; (**F**) Western blot analysis of renal urate transporters. Quantified protein expression levels of (**G**) OAT1, (**H**) ABCG2, (**I**) GLUT9, and (**J**) URAT1. Data are expressed as means ± SD (*n* = 6 mice/group). ^##^ *p* < 0.01 vs. CON group; * *p* < 0.05, ** *p* < 0.01 vs. MOD group.

All animal experiments were conducted following the ethical guidelines approved by the Animal Care and Ethics Committee at the Shandong Academy of Chinese Medicine (approval number: SDZYY20230621002, approval on 25 May 2023). Male Kunming mice (25–28 g) were procured from Vital River Laboratory Animal Technology Co., Ltd. (Beijing, China). The mice were housed under controlled environmental conditions (12 h light/dark cycle, temperature 23 ± 2 °C, relative humidity 50 ± 10%) with free access to standard chow and ultrapure water. According to previous reports [23], as shown in Figure 1A, thirty-six Kunming mice were randomly assigned to six groups (*n* = 6 per group) using a randomized complete block design: control group (CON), HUA model group (MOD), allopurinol-treated group (APL; 5 mg/kg), and three YRO-treated groups (100, 200, and 400 mg/kg) [24]. Except for the CON group, all mice received 300 mg/kg PO (oral) and 300 mg/kg HX (intraperitoneal injection) daily for 21 consecutive days to induce HUA. One hour after the model induction, treatment groups received the corresponding doses of YRO or allopurinol by oral gavage once daily. At the end of the 21-day experimental period, mice were fasted for 12 h before sample collection. Blood, liver, kidney, and intestinal samples were collected for subsequent analysis.

### 2.4. Biochemical and Oxidative Stress Measurements

The levels of serum UA, blood urea nitrogen (BUN), and creatinine (CR) were measured by commercial assay kits (Jiancheng Biotechnology Institute, Nanjing, China). Hepatic xanthine oxidase (XOD) and adenosine deaminase (ADA) activities were also analyzed. Serum lipid parameters, including triglycerides (TG), total cholesterol (TC), low-density lipoprotein cholesterol (LDL-C), and high-density lipoprotein cholesterol (HDL-C), were quantified. Furthermore, the concentrations of renal oxidative indicators, including superoxide dismutase (SOD), malondialdehyde (MDA), and glutathione (GSH), were detected using kits procured from Beyotime Biotechnology Co., Ltd. (Shanghai, China).

### 2.5. Western Blot Analysis

Western blot analysis was conducted according to the method described by Lin et al. [25]. Total proteins were extracted from the kidney and intestine tissues using RIPA lysis buffer (Beyotime Biotechnology Co., Ltd., Shanghai, China). After quantification and denaturation, proteins were separated by SDS-PAGE and transferred onto PVDF membranes. The membranes were blocked and incubated overnight at 4 °C with specific primary antibodies. This was followed by incubation with HRP-conjugated secondary antibodies. Protein bands were visualized using the ECL imaging system and quantified with ImageJ software (version 1.53q, National Institutes of Health, Bethesda, MD, USA). β-Actin or GAPDH was used as the internal loading control to normalize the target protein expression levels.

### 2.6. Histology, Immunohistochemistry (IHC), and Immunofluorescence (IF) Analysis

Kidney and intestinal tissues were fixed in 4% paraformaldehyde, embedded in paraffin, and sectioned into 4 μm slices. For morphological evaluation, hematoxylin and eosin (H&E) was performed. Masson’s trichrome staining was used for fibrosis quantification.

IHC analysis was performed to detect the expression of 8-OHdG, PINK1, and Parkin. Following deparaffinization, rehydration, and antigen retrieval in citrate buffer (pH 6.0), sections were blocked with 3% bovine serum albumin (BSA) and incubated overnight at 4 °C with primary antibodies. After incubation with HRP-conjugated secondary antibodies, staining was visualized using 3,3′-diaminobenzidine (DAB) and counterstained with hematoxylin [26]. IF staining was used to assess the expression of Mfn2 and FIS1. After antigen retrieval and blocking with 5% BSA, sections were incubated overnight at 4 °C with primary antibodies. This was followed by incubation with fluorescently labeled secondary antibodies and counterstaining with DAPI [27]. All stained sections were scanned using a digital slide scanner (KF-PRO-020, Kfbio, Ningbo, China), and quantitative analysis was performed using ImageJ software.

### 2.7. Microbial Community Composition and SCFAs Analysis

Six fecal samples were collected from each of the CON, MOD, and YRO-H groups one day before sacrifice to assess bacterial community composition. Genomic DNA was extracted using the E.Z.N.A.^®^ DNA LQ Kit (Omega Bio-tek, Norcross, GA, USA). The V3-V4 region of the 16S rRNA gene was amplified with primers 338 F and 806R. DNA libraries were constructed using the NEXTFLEX Rapid DNA-Seq Kit (PerkinElmer, Waltham, MA, USA). Sequencing was performed on the Illumina PE300 platform (Illumina Inc., San Diego, CA, USA). Alpha diversity indices, including Coverage, Chao1, ACE, Sobs, Shannon, and Simpson, were calculated to evaluate microbial richness and diversity. Beta diversity was assessed using principal coordinate analysis (PCoA).

For SCFA analysis, fecal samples were extracted with 80% methanol. Derivatization was performed by adding 200 mM 3-nitrophenylhydrazine hydrochloride (3NPH·HCl) and 120 mM 1-ethyl-3-(3-dimethylaminopropyl) carbodiimide hydrochloride (EDC·HCl). The supernatants were analyzed using LC-MS/MS on an ExionLC^TM^ AD system coupled with a QTRAP^®^ 6500+ mass spectrometer (Sciex, Framingham, MA, USA). The SCFAs, including acetic acid, propionic acid, butyric acid, isobutyric acid, valeric acid, and isovaleric acid, were quantified.

### 2.8. Lipid Metabolomics Analysis of Serum

Serum samples were centrifuged, and the supernatant was collected for lipid extraction. Lipids were extracted using methyl tert-butyl ether/methanol (3/1, *v*/*v*) solution containing an internal standard (1000 μL). The lipid extract was then reconstituted in acetonitrile/isopropanol (1/1, *v*/*v*) and centrifuged again. The final supernatant was collected for UPLC-MS/MS analysis, following a modified protocol [28]. Lipid profiling was performed using a SCIEX ExionLC™ AD UPLC-QTRAR system (SCIEX, Framingham, MA, USA) equipped with the Thermo Accucore™ C30 column (2.1 × 100 mm, 2.6 μm; Thermo Fisher Scientific, Waltham, MA, USA). The mobile phases consisted of 60% acetonitrile in water (A) and 10% acetonitrile in isopropanol (B), both supplemented with 0.1% formic acid and 10 mM ammonium formate. The gradient elution program was as follows: 0 min 20% B, 2 min 30% B, 4 min 60% B, 9 min 85% B, and 14 min 90% B with a flow rate of 0.35 mL/min. Mass spectrometry detection was conducted in high-resolution mode with an electrospray ionization (ESI) source. The source temperature was set at 500 °C, with ionization voltages of 5.5 kV in positive mode and 4.5 kV in negative mode.

### 2.9. Statistical Analysis

Statistical analysis and data visualization were performed using GraphPad Prism 8.0 (San Diego, CA, USA). Data are presented as mean ± standard deviation (SD). One-way ANOVA followed by Tukey’s post hoc test was used for comparisons among multiple groups, while comparisons between two groups were performed using independent-sample *t*-tests. A *p*-value of less than 0.05 was considered statistically significant. Statistical significance indicators are specified in the figure legends.

## 3. Results

### 3.1. Fatty Acid Composition of YRO

The fatty acid composition of YRO after methyl esterification is shown in Table S2 and Figure S1. GC-MS analysis, using the area normalization method, identified the methyl esters of 9-octadecenoic acid, 9,12-octadecadienoic acid (Z,Z), and hexadecanoic acid as the primary components. These results indicated that YRO was predominantly composed of unsaturated long-chain fatty acids, which account for 77.11% of the total fatty acids. The most abundant components were oleic acid (42.63%) and linoleic acid (22.36%). Previous studies suggested that diets rich in UFAs could improve lipid metabolism [29], reduce oxidative stress [30], and suppress inflammation [31]. These properties suggest that YRO might offer therapeutic benefits for managing HUA.

### 3.2. YRO Modulated UA Metabolism in HUA Mice

The levels of serum UA in the MOD group (380.31 μmol/L) were significantly higher than in the CON group (37.53 μmol/L) (*p* < 0.01), confirming the successful establishment of the HUA model [32]. YRO treatment at doses of 100, 200, and 300 mg/kg/d reduced serum UA levels in a dose-dependent manner to 102.12, 70.93, and 49.21 μmol/L, respectively (Figure 1C). HUA led to a 63.80% and 45.03% increase in ADA and XOD activities, respectively, compared with the CON group (*p* < 0.01). This significant upregulation demonstrated enhanced catalytic efficiency in UA metabolism within the model group [33]. YRO significantly reduced the activities of ADA and XOD (*p* < 0.05), with the YRO-H group showing decreases of 28.41% and 31.75%, respectively (Figure 1D,E). The expression of renal urate transporters was significantly altered in HUA mice (Figure 1F). The protein expression levels of GLUT9 and URAT1 (urate reabsorption transporters) were elevated, whereas the levels of OAT1 and ABCG2 (urate excretion transporters) were reduced (*p* < 0.05). YRO normalized these transporters levels, showing similar effects to the APL group. These findings indicated that YRO lowered the levels of serum UA partly by the suppression of ADA and XOD activities and modulation of urate transporter expression.

### 3.3. YRO Attenuated HUA-Induced Renal Injury in HUA Mice

**Figure 2:**
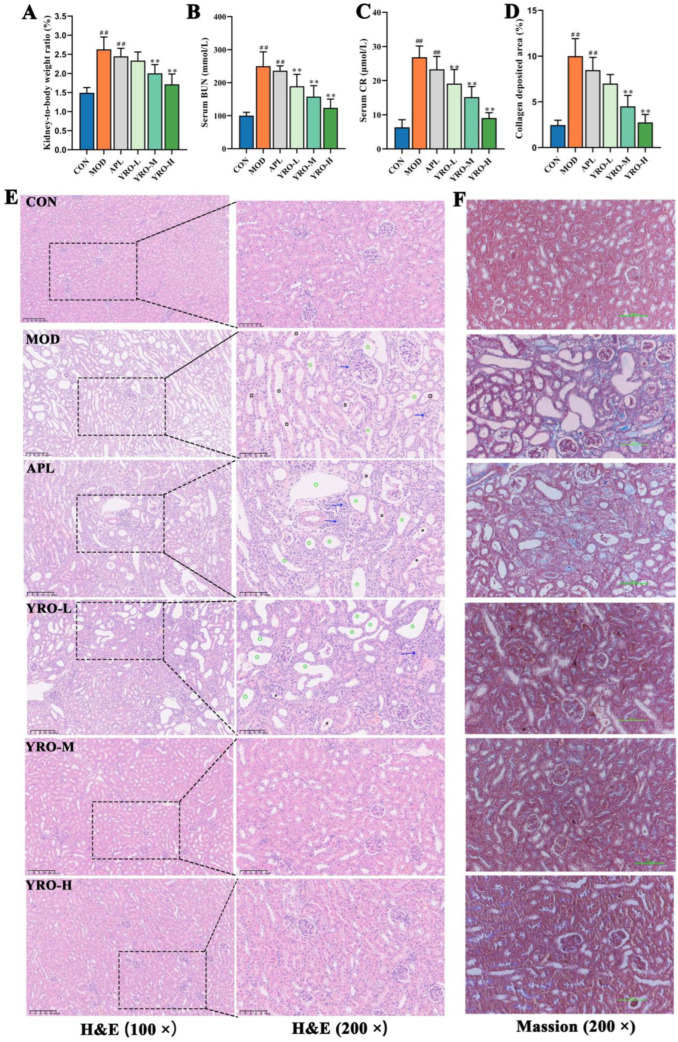
YRO mitigated renal injury in HUA mice. (**A**) Kidney organ coefficients; (**B**) BUN levels; (**C**) CR levels; (**D**) Quantification of the fibrotic area in Masson staining; Morphological assessment of kidney tissues by H&E (**E**) and Masson staining (**F**). Blue arrows, inflammatory cell infiltration; green circles, tubular ectasia; black squares, necrotic tubular epithelial cells. Data are expressed as means ± SD (*n* = 6 mice/group). ^##^ *p* < 0.01 vs. CON group; ** *p* < 0.01 vs. MOD group.

As shown in Figure 1B, body weight gain was significantly slower in the MOD group (*p* < 0.05), while the YRO-treated groups showed no significant difference from the CON group. The kidney-to-body weight ratio was significantly higher in the MOD group, indicating renal impairment. YRO intervention significantly reduced this ratio (*p* < 0.01; Figure 2A), implying improved kidney health. YRO intervention also significantly decreased the levels of CR and BUN (*p* < 0.05; Figure 2B,C), demonstrating its protective effect on renal function. The histopathological analysis further confirmed these findings. H&E staining revealed distinct pathological changes in the MOD group, including renal tubular cavity dilation, glomerular atrophy, and inflammatory cell infiltration. YRO treatment significantly alleviated these pathological changes (Figure 2E). Similarly, Masson’s trichrome staining showed significant renal fibrosis in the MOD group (*p* < 0.05), which was progressively reduced by YRO (Figure 2D,F). These results indicate that YRO mitigates renal damage and fibrosis in HUA mice.

### 3.4. YRO Mitigated Oxidative Stress and Mitochondrial Dysfunction in HUA Mice

**Figure 3:**
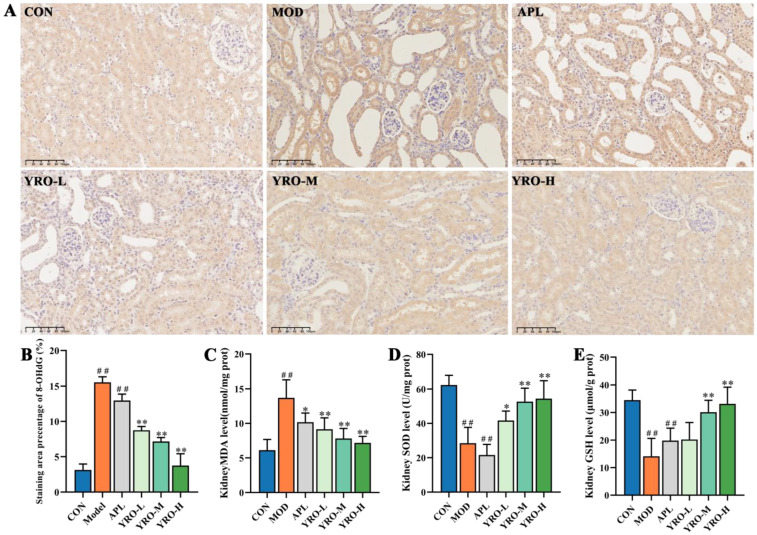
YRO reduced oxidative stress in HUA mice. (**A**) IHC staining of 8-OHdG in renal tubules (200×); (**B**) Quantitative analysis of 8-OHdG expression based on IHC results; (**C**–**E**) Levels of MDA, SOD, and GSH, representing oxidative damage and antioxidant capacity. Data are expressed as means ± SD (*n* = 6 mice/group). ^##^ *p* < 0.01 vs. CON group; * *p* < 0.05, ** *p* < 0.01 vs. MOD group.

Excessive UA induces oxidative stress, leading to cellular and mitochondrial damage [34]. In HUA mice, 8-OHdG levels were significantly elevated, indicating oxidative DNA damage, but YRO treatment markedly reduced its accumulation (*p* < 0.05, Figure 3A,B). Meanwhile, YRO supplementation reversed the elevation in MDA levels, and restored SOD activity and GSH levels (Figure 3C–E), indicating its antioxidant potential.

**Figure 4:**
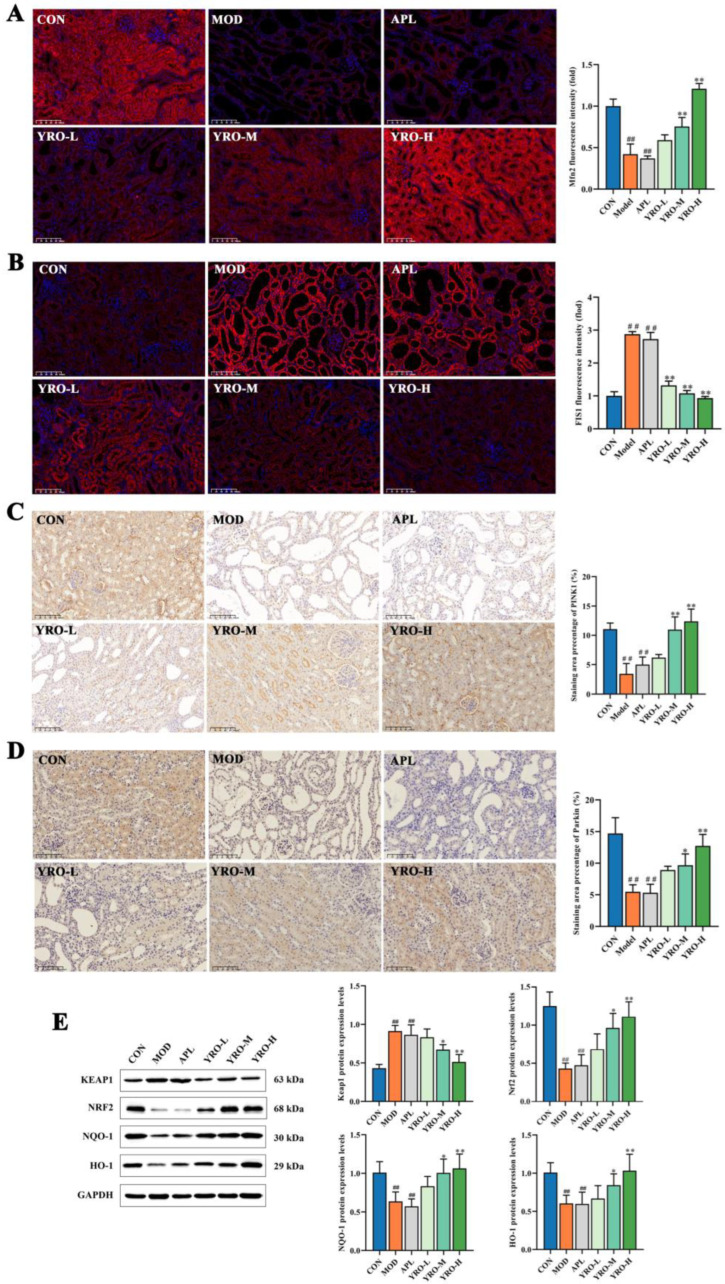
YRO improved mitochondrial function and modulated the oxidative stress pathway in HUA mice. IF staining and fluorescence intensity analysis of (**A**) Mfn2 and (**B**) FIS1; IHC staining and quantification analysis of (**C**) PINK1 and (**D**) Parkin; (**E**) Western blot analysis and protein expression of Keap1, Nrf2, NQO-1, and HO-1. Data are expressed as means ± SD (*n* = 6 mice/group). ^##^ *p* < 0.01 vs. CON group; * *p* < 0.05, ** *p* < 0.01 vs. MOD group.

Given the mitochondrial-rich nature of renal tissues, we further investigated mitochondrial alterations in HUA mice [13]. IF analysis (Figure 4A,B) showed that the expression of Mfn2 was significantly decreased, whereas FIS1 expression was significantly increased in the MOD group (*p* < 0.01). YRO administration upregulated Mfn2 and downregulated FIS1 (*p* < 0.05), restoring mitochondrial fusion-fission balance. IHC analysis revealed that the levels of PINK1 and Parkin were significantly reduced in MOD kidneys (*p* < 0.01), indicating impaired mitophagy. YRO treatment upregulated PINK1 and Parkin, promoting mitochondrial quality control (Figure 4C,D).

To further elucidate the role of YRO in oxidative defense, the expression of key regulators in the Keap1/Nrf2 signaling pathway was examined, as it plays a critical role in maintaining redox balance [35]. Western blot analysis showed that Keap1 expression was significantly increased while Nrf2 levels were markedly suppressed in HUA mice (*p* < 0.01), impairing the antioxidant response. YRO intervention downregulated Keap1 and upregulated Nrf2, leading to the activation of NQO-1 and HO-1, two major antioxidant enzymes (Figure 4E). These findings indicated that YRO enhanced mitochondrial function and antioxidant defenses by modulating the Keap1/Nrf2 pathway.

### 3.5. YRO Enhances Intestinal Barrier Integrity in HUA Mice

**Figure 5:**
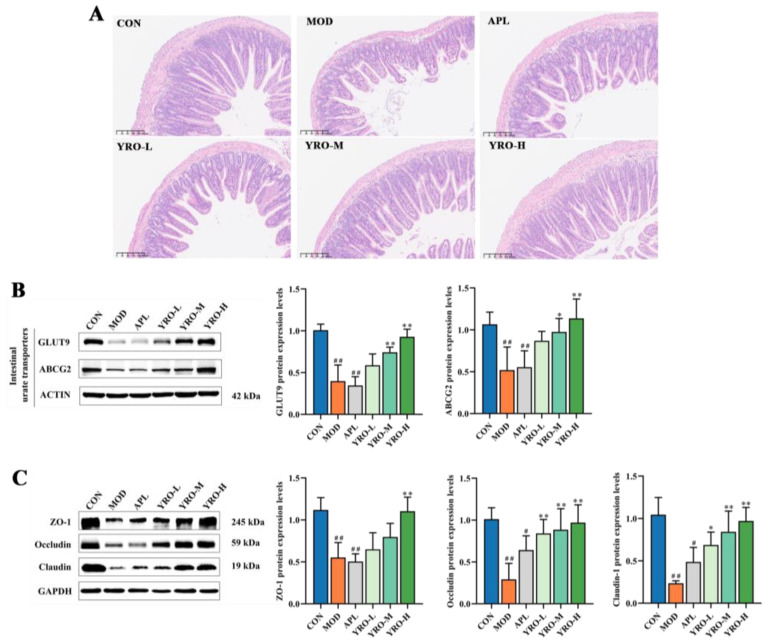
YRO relieved intestinal dysfunction and damage in HUA mice. (**A**) H&E staining of intestine (100×); (**B**) Western blot and quantification analysis of GLUT9 and ABCG2; (**C**) Western blot and quantification analysis of intestine tight protein, including ZO-1, Occludin, and Claudin-1. Data are expressed as means ± SD (*n* = 6 mice/group). ^#^ *p* < 0.05, ^##^ *p* < 0.01 vs. CON group; * *p* < 0.05, ** *p* < 0.01 vs. MOD group.

The intestinal barrier is critical for UA homeostasis by regulating UA excretion and gut permeability [36]. H&E staining showed significant structural changes in the MOD group (Figure 5A). These changes included reduced villus height, disorganized villus structure, and thinning of the muscle layer. Meanwhile, HUA induction led to tight junction proteins (ZO-1, Occludin, and Claudin-1) significant downregulation (*p* < 0.05; Figure 5C), suggesting compromised barrier integrity. The YRO intervention improved the intestinal structure and restored the expression of tight junction protein. Further analysis of intestinal UA transporters showed increased GLUT9 and ABCG2 expression in the YRO group compared with the MOD group (Figure 5B). These changes indicated that YRO promoted intestinal UA excretion, contributing to UA homeostasis.

### 3.6. YRO Modulates Gut Microbiota Diversity and Composition in HUA Mice

**Figure 6:**
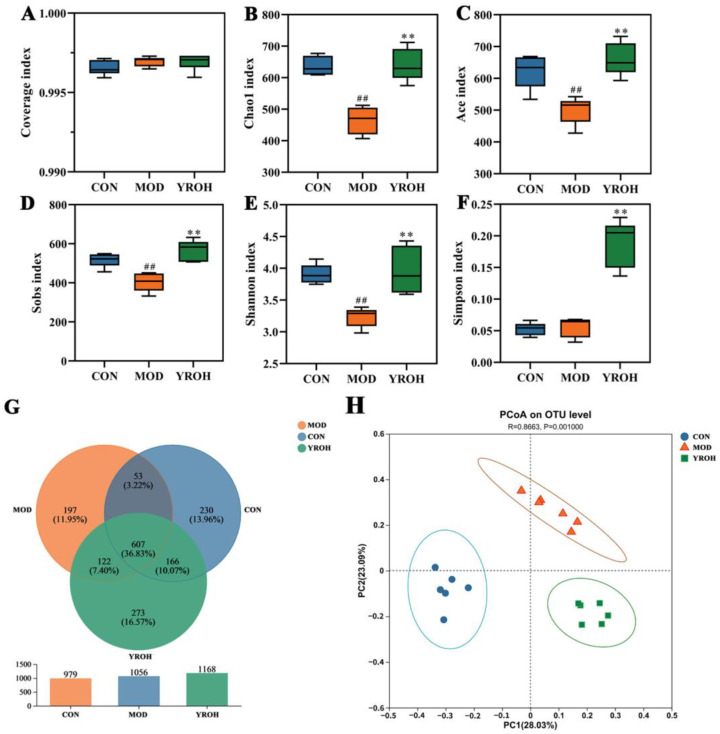
YRO altered gut microbiota structure in HUA mice. α-diversity indices, including (**A**) Coverage; (**B**) Choa1; (**C**) Ace; (**D**) Sobs; (**E**) Shannon; (**F**) Simpson; (**G**) Venn diagram of operational taxonomic units (OTUs); (**H**) PCoA analysis at the genus level. ^##^ *p* < 0.01 vs. CON group; ** *p* < 0.01 vs. MOD group. Note: The total percentage of the pie chart is 100.01% due to rounding.

To investigate the gut microbiota alterations, 16S rDNA sequencing was performed on fecal samples. The coverage index was close to 1, indicating sufficient sequencing depth. HUA mice exhibited significantly reduced microbial diversity and richness, as indicated by lower Chao1, ACE, Shannon, Simpson, and Sobs indices (*p* < 0.05). YRO-H treatment effectively reversed these declines, restoring gut microbiota diversity and richness (Figure 6A–F). The number of operational taxonomic units (OTUs) was higher in YRO-treated mice, with more overlapping OTUs between the YRO and CON groups (Figure 6G). PCoA based on unweighted UniFrac analysis further confirmed that YRO shifted microbial composition, with the YRO-H group clustering separately from the MOD group (Figure 6H).

**Figure 7:**
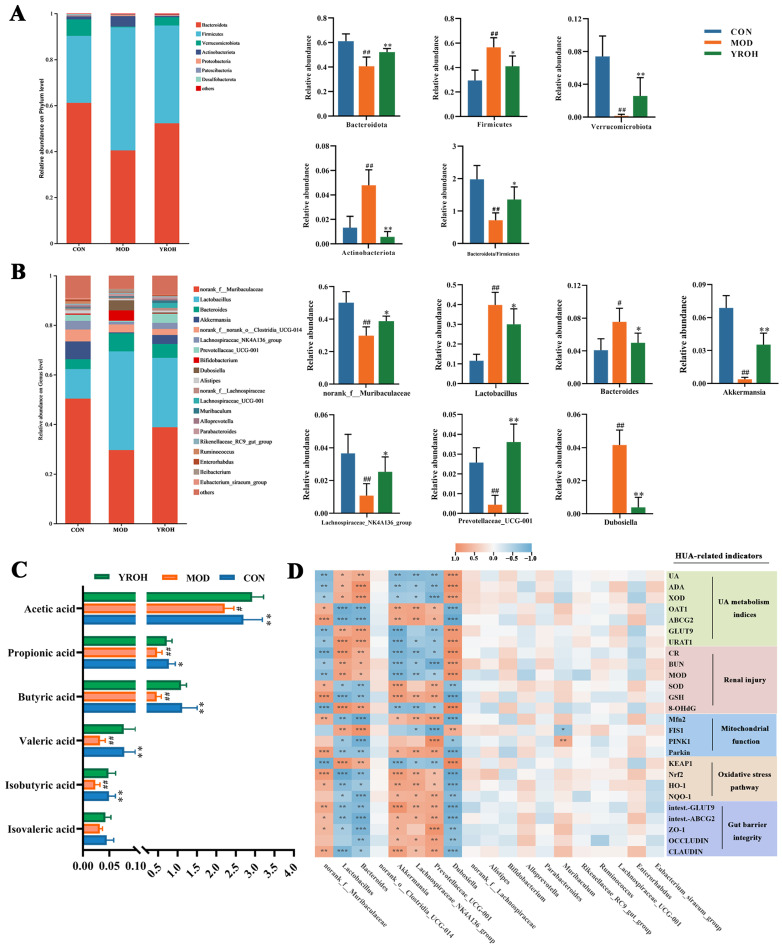
YRO modulated gut microbiota composition and SCFAs production in HUA mice. Microbial community composition and the relative abundance of major gut microbiota (**A**) at the phylum level and (**B**) at the genus level. (**C**) Concentrations of SCFAs. (**D**) Correlation analysis between gut bacteria genera and HUA-related indicators. Data are expressed as means ± SD (*n* = 6 mice/group). ^#^ *p* < 0.05, ^##^ *p* < 0.01 vs. CON group; * *p* < 0.05, ** *p* < 0.01 vs. MOD group. Correlation heatmap significance: * *p* < 0.05, ** *p* < 0.01, *** *p* < 0.001.

At the phylum level, *Firmicutes* and *Bacteroidetes* dominated all groups, followed by *Verrucomicrobia* and *Actinobacteria* (Figure 7A). The MOD group exhibited a significant decrease in the relative abundance of *Bacteroidetes* (40.57%) in comparison to the CON group (61.06%; *p* < 0.05). On the contrary, the relative abundance of *Firmicutes* was markedly increased in the MOD group (53.39%) compared with the CON group (29.44%). YRO-H treatment partially restored these imbalances, adjusting *Bacteroidetes* to 52.19% and *Firmicutes* to 42.78%. YRO-H also mitigated HUA-induced changes in *Verrucomicrobia* and *Actinobacteria*, bringing their abundances closer to those in the CON group.

At the genus level, YRO-H treatment displayed significant alterations in key bacterial genera (Figure 7B). Beneficial genera, including *norank_f__Muribaculaceae*, *Akkermansia*, *Lachnospiraceae_NK4A136_group*, and *Prevotellaceae_UCG-001* were significantly enriched (*p* < 0.05). These genera are associated with gut health, SCFAs production, and inflammation regulation. Furthermore, YRO-H reduced the abundance of *Lactobacillus*, *Bacteroides*, and *Dubosiella*, which were elevated in the MOD group (*p* < 0.05). These findings demonstrate that YRO-H effectively restored microbial diversity, rebalanced the dominant phylum, and promoted the enrichment of beneficial bacterial genera.

### 3.7. YRO Enhanced SCFAs Production in HUA Mice

As shown in Figure 7C, the MOD group exhibited significantly lower SCFA levels, including acetic acid, propionic acid, butyric acid, valeric acid, isobutyric acid, and isovaleric acid, compared with the CON group (*p* < 0.05). YRO-H treatment significantly elevated the concentration of these SCFAs, bringing their levels closer to those observed in the CON group (*p* < 0.05). This restoration demonstrated that YRO-H enhanced SCFAs production, potentially improving gut microbial metabolism in HUA mice.

### 3.8. Correlation Between Intestinal Bacterial Abundances and HUA-Related Biomarkers

Spearman correlation analysis was conducted to explore associations between gut microbiota and physiological indicators (Figure 7D). Beneficial bacteria, including *norank_f__Muribaculaceae*, *Akkermansia*, *Lachnospiraceae_NK4A136_group*, and *Prevotellaceae_UCG-001*, positively correlated with urate excretion transporters, antioxidant capacity, and mitochondrial function markers. These genera were also associated with intestinal barrier integrity, reinforcing their role in gut health. In contrast, *Bacteroides*, *Dubosiella*, and *Lactobacillus* exhibited negative correlations with these physiological markers. Their abundance was linked to increased urate reabsorption, elevated oxidative stress, and compromised gut function. These results indicated the vital role of gut microbiota in mediating the protective effects of YRO against HUA.

### 3.9. YRO Attenuated Lipid Metabolism Disorders in HUA Mice

#### 3.9.1. YRO Modulated Serum Lipid Profile in HUA Mice

**Figure 8:**
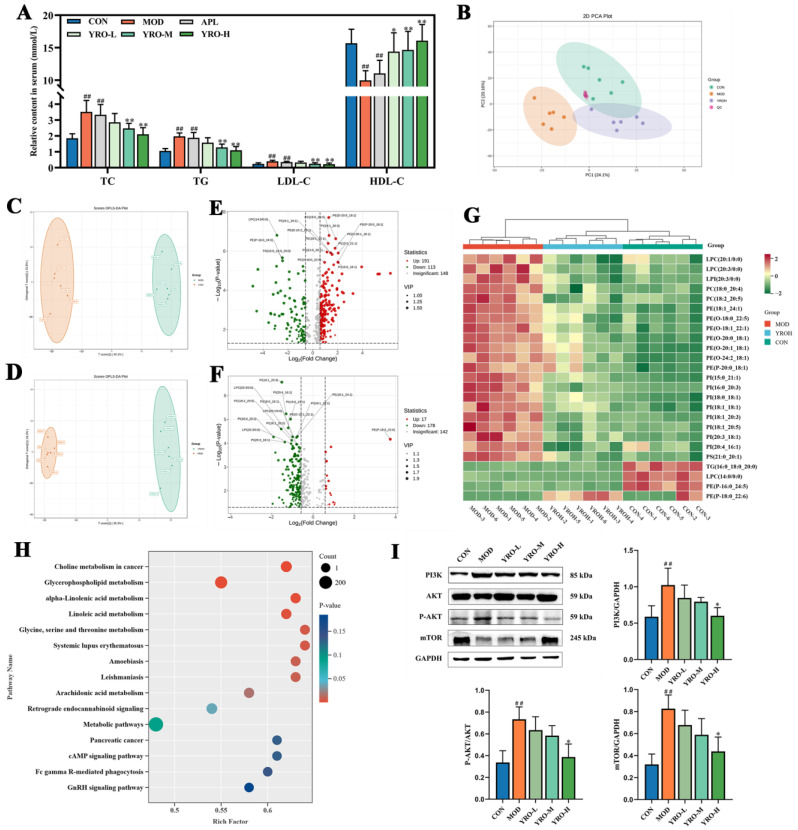
YRO attenuated serum lipid metabolism disorders in HUA mice. (**A**) Serum concentrations of TC, TG, LDL-C, and HDL-C; (**B**) PCA score plot of CON, MOD, YROH, and QC groups; OPLS-DA score plots of (**C**) CON vs. MOD and (**D**) YROH vs. MOD; Volcano maps and top 15 differential lipids of (**E**) CON vs. MOD and (**F**) YROH vs. MOD; (**G**) Heatmap of differential lipid metabolites in the CON, MOD, and YROH groups; (**H**) KEGG pathway annotation between the MOD and YROH groups; (**I**) Modulation of YRO on PI3K/AKT/m-TOR pathway protein expression in the kidney. ^##^ *p* < 0.01 vs. CON group; * *p* < 0.05, ** *p* < 0.01 vs. MOD group.

Compared with the CON group, serum TC, TG, and LDL-C levels were significantly elevated in the MOD group (*p* < 0.01; Figure 8A), while HDL-C levels were reduced. YRO treatment significantly reversed these changes (*p* < 0.05), restoring lipid levels closer to normal. In the analysis of serum lipid metabolism, principal component analysis (PCA) demonstrated a clear separation between the MOD and YROH groups, with the YRO-H cluster closer to the CON group (Figure 8B). Orthogonal partial least squares discriminant analysis (OPLS-DA) further confirmed distinct metabolic profiles among the three groups (Figure 8C,D), indicating that YROH intervention modulated serum lipid metabolism in HUA mice.

Lipid metabolite alterations were identified using VIP > 1 and *p* < 0.05. The top 15 differentially abundant lipids were selected for both CON vs. MOD and YROH vs. MOD comparisons (Figure 8E,F). Phosphatidylethanolamine (PE) and phosphatidylinositol (PI) were the most significantly altered lipid classes in the MOD group, showing marked increases compared to the CON group (Table S3). YROH treatment reversed these changes, restoring PE and PI levels closer to normal (Figure 8G). Additionally, five specific lipid species were significantly altered in both comparisons, including PE (18:1_24:1), PI (15:1_21:1), PI (18:0_18:1), PI (18:1_18:1), and PI (18:1_20:3). Their levels were elevated in HUA mice but were restored by YRO treatment, suggesting a potential role in lipid homeostasis.

#### 3.9.2. KEGG Pathway Enrichment Analysis

KEGG enrichment analysis identified the key metabolic pathways between the MOD and YROH groups (Figure 8H). YRO-H treatment significantly enriched pathways related to lipid metabolism, particularly glycerophospholipid metabolism and α-linolenic acid metabolism. Notably, choline metabolism in cancer exhibited the strongest enrichment trend, reflecting its involvement in choline utilization and cellular metabolic regulation.

#### 3.9.3. YRO Modulated the PI3K/AKT/mTOR Signaling Pathway

As choline metabolism is closely linked to the PI3K/AKT/mTOR signaling pathway, western blot analysis was conducted to evaluate the regulatory effect of YRO on its activation (Figure 8I). In HUA mice, PI3K, phosphorylated AKT (P-AKT), and mTOR levels were significantly elevated, indicating activation of this pathway. Overactivation of this pathway is associated with lipid metabolism dysfunction, oxidative stress, and cellular damage in HUA [37]. YRO intervention significantly downregulated PI3K, P-AKT, and mTOR expression, with the strongest inhibition observed in the YRO-H group. These findings suggested that YRO may mitigate oxidative stress in HUA mice by suppressing PI3K/AKT/mTOR activation.

## 4. Discussion

The increasing prevalence of HUA, driven by dietary and lifestyle changes, is a growing public health concern. HUA-related complications, including kidney dysfunction and intestinal barrier impairment, are closely linked to oxidative stress and metabolic disturbances. YRO is abundant in UFAs and has the potential to alleviate oxidative stress and support organ function. This study explored the mechanisms underlying the protective effects of YRO in HUA, focusing on UA metabolism, oxidative stress regulation, intestinal barrier integrity, and lipid homeostasis.

UA homeostasis depends on a dynamic balance between synthesis and excretion [38]. ADA and XOD are key enzymes involved in the purine degradation pathway, ultimately leading to UA formation [39]. In this study, YRO suppressed both ADA and XOD activity, reflecting a significant reduction in endogenous UA production. UA clearance is tightly regulated by renal and intestinal transport systems. In the kidney, reabsorption transporters GLUT9 and URAT1 were abnormally upregulated in HUA mice, leading to excessive UA retention and renal overload [40]. YRO intervention effectively downregulated these transporters while restoring the expression of the excretory transporters OAT1 and ABCG2. Notably, YRO also enhanced intestinal ABCG2 and GLUT9 expression, supporting an alternative route for UA elimination. By simultaneously reducing UA biosynthesis and enhancing renal and intestinal excretion, YRO comprehensively improved UA metabolism and alleviated systemic urate burden.

Excessive UA accumulation in the kidney disrupts renal function and exacerbates oxidative stress-related injury [41]. Histological analysis further confirmed that YRO preserved tubular integrity, reduced renal fibrosis, and improved kidney morphology. The decreases in CR and BUN levels provided additional evidence of improved renal function. Mitochondria, as the primary site of cellular energy metabolism, are highly vulnerable to UA-induced oxidative stress [42]. In HUA mice, disrupted mitochondrial dynamics, characterized by decreased Mfn2 and increased FIS1 expression, indicated excessive mitochondrial fragmentation [43]. Meanwhile, Keap1/Nrf2 signaling, a crucial antioxidant defense pathway, was suppressed, further exacerbating oxidative damage [44]. YRO administration restored mitochondrial fusion-fission balance, enhanced mitophagy via PINK1/Parkin activation, and promoted mitochondrial quality control.

Additionally, YRO alleviated Keap1-mediated inhibition of Nrf2, facilitating its nuclear translocation and upregulating antioxidant enzymes (NQO-1 and HO-1). Thereby reinforcing redox homeostasis and mitigating oxidative injury. Reactive oxygen species (ROS) contribute to UA-induced oxidative damage and are known activators of the NLRP3 inflammasome, a key driver of inflammation in HUA [45,46]. Notably, YRO reduced mitochondrial dysfunction and enhanced antioxidant defense, which may indirectly suppress NLRP3 activation. These observations align with emerging evidence linking redox balance to inflammasome regulation and a potential mechanism that warrants further investigation.

The kidneys and the intestine jointly regulated UA excretion, with intestinal excretion compensating for renal impairment [47]. In the HUA process, excessive UA accumulation damaged the intestinal barrier, increasing permeability and inflammation, which further disrupted UA elimination [48]. Tight junction proteins (ZO-1, Occludin, and Claudin-1) are essential for maintaining epithelial integrity, and their reduced expression in HUA mice indicated barrier dysfunction [49]. YRO effectively restored the expression of these tight junction proteins, strengthening the intestinal barrier and facilitating UA excretion.

The gut microbiota has always been proven to be a vital regulator in UA metabolism and gut functional homeostasis [27,50]. In this study, correlation analysis demonstrated that gut microbiota composition was closely associated with urate excretion transporters, antioxidant capacity, and mitochondrial function markers, reinforcing its role in HUA progression and metabolic regulation. HUA mice exhibited significant microbial dysbiosis, characterized by a decreased Bacteroidetes-to-Firmicutes ratio (Figure 7A), an established marker of metabolic imbalance [51]. This dysbiosis was associated with an increase in pathogenic bacteria and a reduction in beneficial microbes, likely exacerbating intestinal barrier dysfunction and UA accumulation. Several studies have linked HUA-associated dysbiosis to an overabundance of *Bacteroides* and *Dubosiella*, which trigger gut-derived intestinal permeability disruption and oxidative stress [52,53]. In agreement with these findings, HUA mice in this study displayed significantly increased levels of *Bacteroides* and *Dubosiella*. This shift may contribute to enhanced UA reabsorption, further worsening HUA symptoms. 

YRO intervention reduced the abundance of these pathogenic bacteria, suggesting that it may alleviate oxidative stress and gut metabolic dysfunction by modulating microbial composition. At the same time, YRO increased the abundance of beneficial bacteria, particularly *Lachnospiraceae_NK4A136_group* and *Akkermansia*, which have been reported to play a role in SCFAs production and metabolic stability [54,55]. SCFAs, including acetate, propionate, and butyrate, are essential for intestinal barrier integrity, immune modulation, and energy metabolism [56]. Previous studies reported that HUA mice often exhibit reduced SCFA levels, which may impair intestinal function and metabolic regulation. Our results confirmed this trend, as SCFA concentrations were significantly lower in HUA mice but were restored following YRO administration, reinforcing its role in *Lachnospiraceae_NK4A136 _group* and *Akkermansia* regulation. Another notable shift was observed in *Lactobacillus*, which was significantly elevated in HUA mice. This genus plays a key role in purine metabolism, and its increase may represent an adaptive response to excessive purine accumulation [57,58]. YRO supplementation normalized *Lactobacillus* levels, indicating a potential restoration of purine metabolism homeostasis and a reduction in UA synthesis.

Meanwhile, emerging human studies have consistently shown that HUA is associated with increased *Bacteroides* and *Dubosiella*, and other pathogenic bacteria, alongside reductions in SCFA-producing and barrier-supporting bacteria such as *Akkermansia* and *Lachnospiraceae* [59,60]. These microbial patterns are consistent with those reported in HUA patients, suggesting that YRO-induced microbiota remodeling may have translational relevance. Further studies incorporating fecal microbiota transplantation (FMT) from YRO-treated mice are needed to determine whether gut microbiota alterations directly mediate the observed benefits.

Lipid metabolism is fundamental to energy homeostasis, membrane stability, and cellular signaling, and its disruption is frequently associated with metabolic disorders, including HUA [61]. Glycerophospholipids, particularly PI and PE, are key regulatory lipids involved in these processes [62]. In this study, lipidomic analysis revealed significant alterations in PI and PE levels in HUA mice. YRO administration restored PI and PE levels and indicated its ability to stabilize lipid metabolism. Further pathway analysis using KEGG enrichment demonstrated that PI and PE metabolism were closely associated with the PI3K/AKT/mTOR signaling pathway. PI directly activates PI3K, modulating downstream metabolic and oxidative responses.

Previous studies have demonstrated that PI3K/AKT/mTOR signaling is abnormally activated in HUA, contributing to renal fibrosis [63]. The findings of this study are consistent with these reports, confirming that HUA-induced PI3K/AKT/mTOR hyperactivation is associated with kidney injury. However, in addition to its role in renal fibrosis, this pathway is also involved in mitophagy, apoptosis regulation, and antioxidant responses [64]. Further analysis revealed that PI3K/AKT/mTOR hyperactivation in HUA was accompanied by impaired Nrf2 signaling. The YRO intervention effectively reversed these abnormalities. It not only restored Nrf2 activation, leading to enhanced antioxidant enzyme expression and reduced oxidative burden, but also suppressed PI3K/AKT/mTOR signaling, mitigating oxidative stress-related damage. These findings suggested that YRO regulated lipid metabolism and oxidative stress responses, thereby contributing to its protective effects in HUA.

Recent studies have identified several functional oils with hypouricemic potential, including *Sonneratia apetala* seed oil (SSO), cassia oil, and celery seed oil [24,65,66]. Among them, SSO primarily consists of linoleic acid (69.6%) and oleic acid (5.8%), exerting dual effects via XOD inhibition and modulation of urate transporter expression, while also attenuating renal oxidative damage through the Keap1–Nrf2 axis [24]. Similarly, YRO contains substantial amounts of oleic acid (42.63%) and linoleic acid (22.36%), which may synergistically contribute to its multi-target bioactivity. Future studies will investigate whether individual fatty acids, or their combinations, are responsible for the observed effects, supporting mechanistic dissection and targeted formulation development. In addition to restoring renal urate transporter balance and antioxidant, YRO reversed HUA-induced mitochondrial fragmentation and upregulation of antioxidant enzymes (HO-1 and NQO-1), indicating improved mitochondrial quality control and redox balance. These were essential aspects of renal resilience under urate stress. Meanwhile, YRO uniquely enhanced intestinal urate excretion and improved lipid disorder, further distinguishing its mode of action from previously studied oils. Although this study focused on the lipid composition of coix seed, the plant also contains other bioactive components, including flavonoids [67]. Notably, genistein has been shown to reduce risk markers of metabolic syndrome [68]. Given the increasing interest in plant-based dietary strategies for metabolic regulation, future studies may explore whether coix seed oil, in combination with other natural compounds, could provide added benefits for managing HUA and related diseases.

## 5. Conclusions

YRO effectively ameliorated HUA-related symptoms in mice and mitigated HUA-induced oxidative damage in the kidney. Importantly, YRO restored microbial homeostasis and improved lipid metabolism. This study revealed the UA-lowering role of YRO on hyperuricemia and its underlying mechanisms involving renal and intestinal regulation. While these findings are promising, further studies, including clinical trials, are warranted to validate their safety and efficacy in humans and to explore their potential as a nutraceutical intervention for hyperuricemia.

## Data Availability

The data sets generated and/or analyzed during the current study are either shown in the manuscript and Supplementary Materials or available from the corresponding author on reasonable request.

## Supplementary Materials

The following supporting information can be downloaded at: https://www.mdpi.com/article/10.3390/nu17101679/s1, Figure S1: GC-MS total ions chromatogram of methyl esterification of YRO; Table S1: Antibody information for Western blotting, IHC, and IF; Table S2: Component and distribution of fatty acids on YRO; Table S3: Altered profiles of key lipid metabolites following YROH treatment in HUA.
