# Supplementary material for: Coix Seed Oil Alleviates Hyperuricemia in Mice by Ameliorating Oxidative Stress and Intestinal Microbial Composition
Source: Nutrients. 2025 May 15;17(10):1679. doi: 10.3390/nu17101679 (PMC12114407; doi:10.3390/nu17101679)
Supplement: Supplementary file 1 [file nutrients-17-01679-s001.zip › nutrients-3619722-supplementary.pdf]

Supplementary Materials

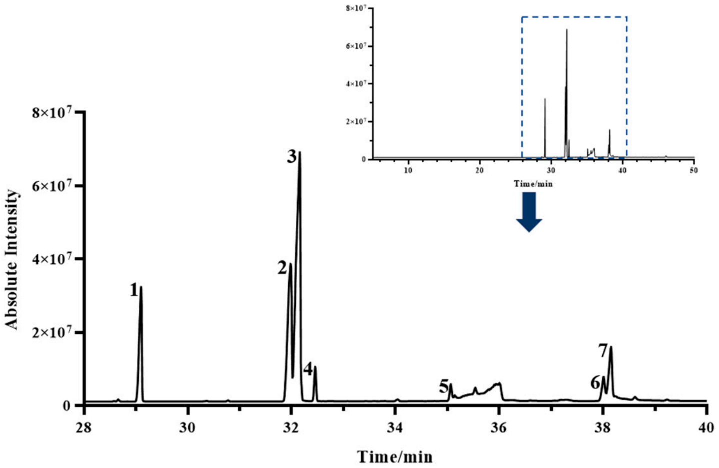

Figure S1 GC-MS total ions chromatogram of methyl esterification of YRO

Table S1 Antibody information for Western blotting, IHC, and IF

Antibody	Abbreviations	Manufacture	Dilution
Urate Transporter 1	URAT1	Proteintech	1: 1000
Glucose Transporter 9	GLUT9	Proteintech	1:500
Organic Anion Transporter 1	OAT1	Proteintech	1:800
ATP-Binding Cassette Subfamily G Member 2	ABCG2	Cell Signaling Technology	1:1000
Glyceraldehyde 3-Phosphate Dehydrogenase	GAPDH	Cell Signaling Technology	1:1000
Beta-Actin	β-ACTIN	ZSGB-Bio	1:1500
8-Hydroxy-2'-deoxyguanosine	8-OHdG	Bioss	1:100
PTEN-Induced Kinase 1	PINK1	Proteintech	1:1000
Parkinson Protein	Parkin	Proteintech	1:100
Mitofusin 2	Mfn2	Bioss	1:100
Fission 1	FIS1	Proteintech	1:200
Kelch-Like ECH-Associated Protein 1	Keap1	Cell Signaling Technology	1:1000
Nuclear Factor Erythroid 2-Related Factor 2	Nrf2	Cell Signaling Technology	1:1000
Heme Oxygenase 1	HO-1	Cell Signaling Technology	1:1000
NAD(P)H Quinone Dehydrogenase 1	NQO-1	Cell Signaling Technology	1:1000

Table S2 Component and distribution of fatty acids on YRO

No.	Retention time (min)	Fatty Acid Methyl Esters	Relative content (%)	Fatty acid	Fatty acid molecular formula
1	29.10	Hexadecanoic acid methyl ester	12.16%	Palmitic acid	$C_{16}H_{32}O_2$
2	31.98	9,12-Octadecadienoic acid (Z, Z) methyl ester	22.36%	Linoleic acid	$C_{18}H_{32}O_2$
3	32.16	9-Octadecenoic acid methyl ester	42.63%	Oleic acid	$C_{18}H_{34}O_2$
4	32.46	Methyl stearate methyl ester	2.66%	Stearic acid	$C_{18}H_{36}O_2$
5	35.07	Glycidyl palmitate methyl ester	1.56%	Palmitic acid	$C_{16}H_{32}O_2$
6	38.02	9,11-Octadecadienoic acid (E,E) methyl ester	3.08%	Conjugated linoleic acid	$C_{18}H_{32}O_2$
7	38.16	Glycidyl palmitoleate methyl ester	9.04%	Palmitoleic acid	$C_{16}H_{30}O_2$

Table S3. Altered profiles of key lipid metabolites following YROH treatment in HUA

No.	Metabolite	Molecular formula	VIP score	Trend change	
				MOD *vs.* CON	YRO-H *vs.* MOD
1	LPC(20:1/0:0)	$C_{28}H_{56}NO_7P$	1.133	↑#	↓**
2	LPC(20:3/0:0)	$C_{28}H_{52}NO_7P$	1.539	↑##	↓**
3	LPI(20:3/0:0)	$C_{29}H_{51}O_{12}P$	1.564	↑##	↓**
4	PC(18:0_20:4)	$C_{46}H_{84}NO_8P$	1.528	↑##	↓**
5	PC(18:2_20:5)	$C_{46}H_{78}NO_8P$	1.474	↑##	↓**
6	PE(18:1_24:1)	$C_{47}H_{90}NO_8P$	1.900	↑##	↓**
7	PE(O-18:0_22:5)	$C_{45}H_{82}NO_7P$	1.657	↑##	↓**
8	PE(O-18:1_22:1)	$C_{45}H_{88}NO_7P$	1.743	↑##	↓**
9	PE(O-20:0_18:1)	$C_{43}H_{86}NO_7P$	1.903	↑##	↓**
10	PE(O-20:1_18:1)	$C_{43}H_{84}NO_7P$	1.881	↑##	↓**
11	PE(O-24:2_18:1)	$C_{47}H_{90}NO_7P$	1.895	↑##	↓*
12	PE(P-20:0_18:1)	$C_{43}H_{84}NO_7P$	1.778	↑##	↓*
13	PI(15:0_21:1)	$C_{45}H_{85}O_{13}P$	1.953	↑##	↓**
14	PI(16:0_20:3)	$C_{45}H_{81}O_{13}P$	1.725	↑##	↓**
15	PI(18:0_18:1)	$C_{45}H_{85}O_{13}P$	1.930	↑##	↓**
16	PI(18:1_18:1)	$C_{45}H_{83}O_{13}P$	1.905	↑##	↓**
17	PI(18:1_20:3)	$C_{47}H_{83}O_{13}P$	1.837	↑##	↓**
18	PI(18:1_20:5)	$C_{47}H_{79}O_{13}P$	1.661	↑##	↓**
19	PI(20:3_18:1)	$C_{47}H_{83}O_{13}P$	1.800	↑##	↓**
20	PI(20:4_16:1)	$C_{45}H_{77}O_{13}P$	1.058	↑##	↓**
21	PS(21:0_20:1)	$C_{47}H_{90}NO_{10}P$	1.710	↑##	↓**
22	TG(16:0_18:0_20:0)	$C_{57}H_{110}O_6$	1.697	↓##	--
23	LPC(14:0/0:0)	$C_{22}H_{46}NO_7P$	1.533	↓##	--
24	PE(P-16:0_24:5)	$C_{45}H_{80}NO_7P$	1.460	↓##	--
25	PE(P-18:0_22:6)	$C_{45}H_{78}NO_7P$	0.416	--	↑**

#*p* < 0.05 *vs* CON group; ##*p* < 0.01 *vs* CON group; **p* < 0.05 *vs* MOD group; ***p* < 0.01 *vs* MOD group. (↑): up-regulated and (↓): down-regulated.
